# Recent Advances in Organic Light-Emitting Diodes Based on Pure Organic Room Temperature Phosphorescence Materials

**DOI:** 10.3389/fchem.2019.00305

**Published:** 2019-05-07

**Authors:** Ge Zhan, Zhiwei Liu, Zuqiang Bian, Chunhui Huang

**Affiliations:** Beijing National Laboratory for Molecular Sciences, Beijing Engineering Technology Research Centre of Active Display, College of Chemistry and Molecular Engineering, Peking University, Beijing, China

**Keywords:** organic light-emitting diodes, room-temperature phosphorescence, external quantum efficiency, lifetime, optoelectronic functional devices

## Abstract

Pure organic room temperature phosphorescence (RTP) materials have attracted extensive attention in recent years due to their unique characteristics, such as flexible design method, low toxicity, low cost, as well as the ease of production at scale. The involvement of triplet state and direct radiative transition from the triplet state show that RTP materials have great potential as a new generation emitter in organic light-emitting diodes (OLEDs). Based on the mechanism of phosphorescence, various methods have been developed to achieve RTP emissions in the crystal state. However, the observation of RTP in the thin film state is much more difficult to achieve because of the lower degree of rigidity and suppression of the non-radiative transition. In this mini-review, molecular design strategies developed to achieve RTP emissions and their application in OLEDs are summarized and discussed. The conclusion and outlook point to great potential as well as the challenges for the continued study of pure organic RTP materials-based OLEDs.

## Introduction

The early stage of organic light-emitting diodes (OLEDs) are based on fluorescent materials (Tang and Vanslyke, 1987), which could not utilize the triplet excitons that accounted for 75% of the total excitons (Baldo et al., 1999), and caused incomplete energy utilization and low device efficiency. In 1998, Ma et al. (1998) and Baldo et al. (1998) introduced osmium complex and platinum complex as luminescent materials into OLEDs, which increased the theoretical maximum internal quantum efficiency (IQE) of the device from 25% of the fluorescent material to 100% of the phosphorescent material. So far, phosphorescent OLEDs have achieved great success and has even been applied in commercial devices, such as mobile phones, televisions, and so on.

However, the noble metals contained in phosphorescent complexes are expensive, low in abundance and toxic, which restricts the further development and popularization of OLEDs. Therefore, metal-free luminescent materials have attracted increased interests in OLEDs, among which thermally activated delayed fluorescent (TADF) materials and pure organic room temperature phosphorescence (RTP) materials are successively introduced into OLEDs as emitters, while the OLEDs also showed a theoretical maximum IQE up to 100%.

Different from TADF materials, which have been demonstrated great success in OLEDs, the application of pure organic RTP materials in OLEDs is still in its initial stage, because high efficiency and short-lived RTP molecules suitable for OLEDs are rare. Nevertheless, RTP materials will provide more possibilities for high performance OLEDs and deserve to be explored further. This mini-review starts with an introduction to basic concepts such as RTP and OLEDs, and then discusses representative work on the electroluminescence study of pure organic RTP materials as well as the reported pure organic RTP materials potentially using in fabricating OLEDs. Finally, the potential and challenges of the study of electroluminescence on pure organic RTP materials are summarized.

## Basic Principles for RTP

**Figure 1:**
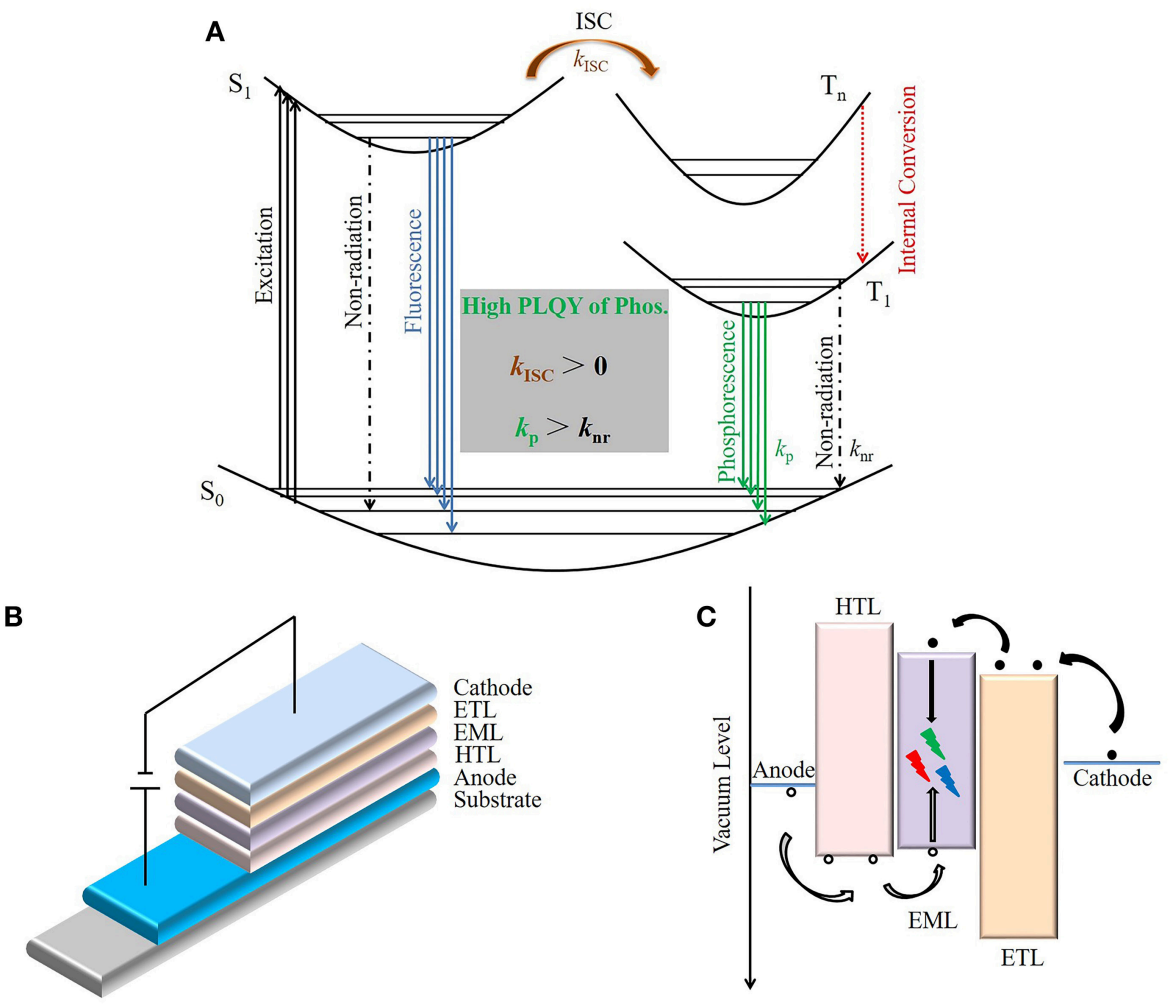
Schematic diagram of OLEDs based on RTP materials. **(A)** Schematic Jablonski diagram of photoluminescence for RTP materials. **(B)** The typical structure of three-layer OLEDs. **(C)** The schematic injection, transport, and recombination process of holes (black circles) and electrons (black dots) in OLEDs.

In general, the production of phosphorescence in pure organics involves two necessary processes: (i) intersystem crossing (ISC) from the lowest excited singlet state (S_1_) to a triplet state (T_n_) and (ii) radiative transition from the lowest excited triplet state (T_1_) to the ground state (S_0_) (Figure 1A). However, the excited triplet state can only be generated by ISC process from an excited singlet state (Reineke and Baldo, 2014). Therefore, *k*_ISC_ > 0 is a necessary condition for generating phosphorescence emission, where *k*_ISC_ is the ISC rate (Hirata, 2017). Only the energy level and electronic configuration determine *k*_ISC_. The ISC process can be accelerated by a small energy gap between S_1_ and T_1_ (ΔE_ST_). Experiments have shown that the ISC and reverse ISC (RISC) process are both accelerated when ΔE_ST_ is extremely small (< 100 meV), and TADF emission can be obtained under this condition (Uoyama et al., 2012). However, TADF showed a different photophysical process to phosphorescence, as TADF contains both a prompt and delayed radiative transition from S_1_ to S_0_, while phosphorescence is a radiative transition from T_1_ to S_0_. The effect of electronic configuration on ISC has been confirmed by El-Sayed (Kalyanasundaram et al., 1977). He found that the spin-orbit coupling could be promoted by mixing different electronic configuration singlet and triplet states, such as (π, π^*^) and (n, π^*^). In addition, the heavy atom effect is also widely used to accelerate the *k*_ISC_ process (Plummer et al., 1993). Therefore, introducing n electrons containing atoms such as O and N, and heavy atoms like Br and I, are strategies frequently used to design efficient RTP materials.

Due to a longer lifetime, the excited triplet state can be easily quenched under ambient conditions (Schulman and Parker, 1977). The second necessary condition for obtaining high efficiency phosphorescence is *k*_P_ > *k*_nr_, where *k*_P_ and *k*_nr_ are a radiative and non-radiative transition rate from T_1_ to S_0_, respectively. The non-radiative process can be divided into external losses caused by the interaction with environmental conditions and intramolecular losses (Liu et al., 2016). At room temperature, *k*_P_ is generally less than *k*_nr_ in pure organic compounds, which is the main reason for the low photoluminescence quantum yield (PLQY) of pure organic RTP materials. Therefore, suppressing non-radiative transitions may be the most important and challenging part of achieving effective RTP in pure organic materials.

## Brief Introduction to OLEDs

A typical OLEDs (Figure 1B) includes a hole transport layer (HTL), an emitting layer (EML), an electron transport layer (ETL), an anode, and a cathode. In addition, a hole injection layer (HIL) and an electron injection layer (EIL) are widely used to reduce the carrier injection barrier from the electrode to the organic layer, while a hole blocking layer (HBL) and an electron blocking layer (EBL) are usually used to effectively confine the hole and electron within the EML. Based on the working mechanism (Figure 1C), the external quantum efficiency (EQE) of OLEDs could be deduced as EQE = η_e•h_ × η_PL_ × η_exciton_ × η_out_, where η_e•h_ is the recombination efficiency of injected holes and electrons, η_PL_ is the intrinsic photoluminescence efficiency, i.e., PLQY of the EML, η_exciton_ is the radiative exciton ratio, and η_out_ is the light out-coupling efficiency. Since the theoretical η_exciton_ and η_out_ are 100% and ca. 20% respectively, the challenge for RTP based OLEDs is to achieve high PLQY in a thin film.

## OLEDs Based on Pure Organic RTP Materials

Though pure organic RTP materials have great potential as emitters in OLEDs, there are only a few examples of the study of electroluminescence in pure organic RTP materials, since most reported pure organic RTP materials showed low PLQY and a long excited lifetime (Kabe and Adachi, 2017), which is not suitable for fabricating high efficiency OLEDs.

**Figure 2:**
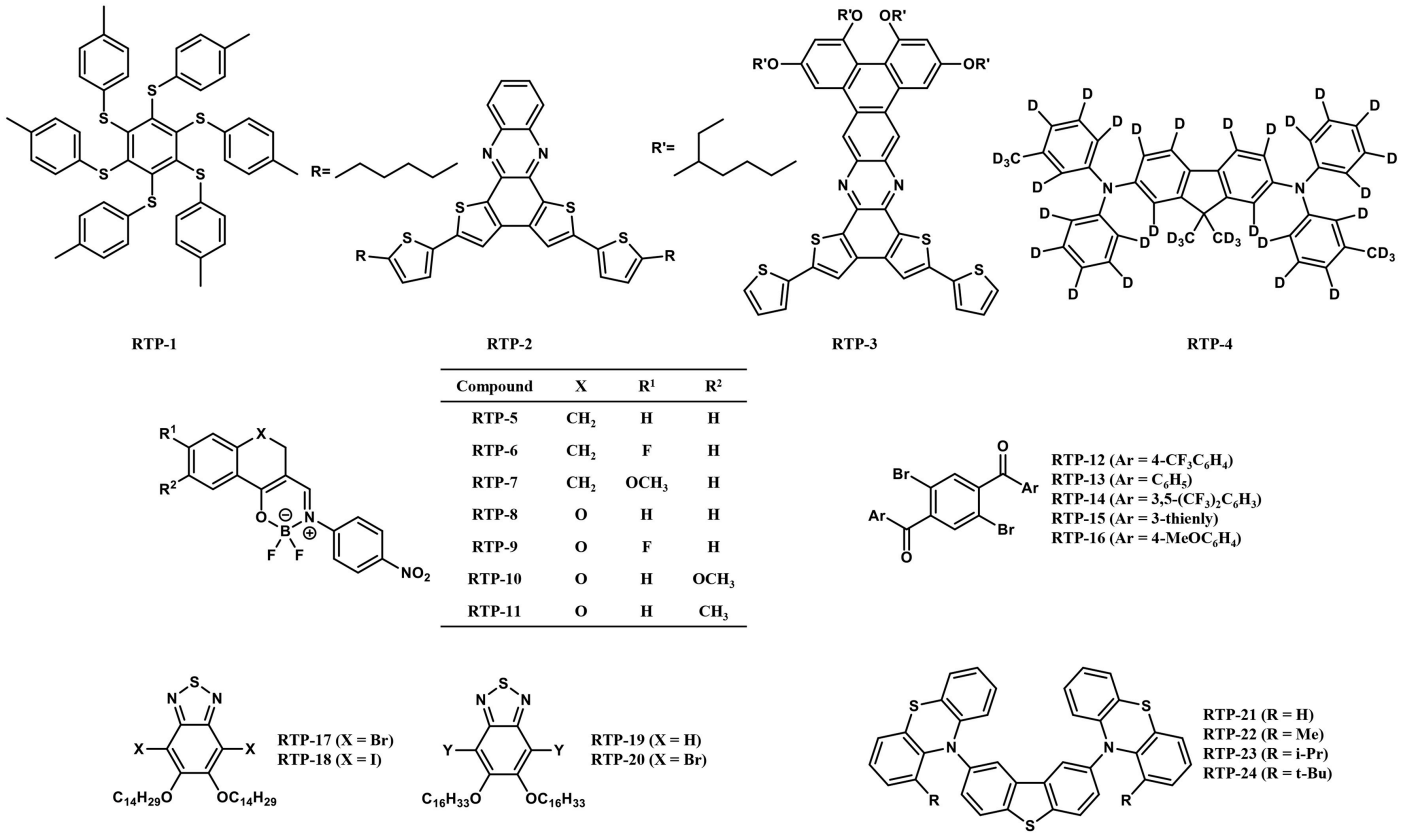
Chemical structure of RTP materials used or potential to be used in OLEDs.

In 2013, Bergamini et al. synthesized **RTP-1** (Figure 2) consisting of a hexathio-benzene core and peripheral tolyl substituents (Bergamini et al., 2013). The compound showed outstanding phosphorescence in solid state at room temperature, while no luminescence was observed in solution. The authors attributed the luminescence behavior to a rigid environment and limited conformational migration of the tolyl substituent, which suppresses the non-radiative deactivation process from T_1_. Since the compound showed a high PLQY up to 80% in solid powder, the authors applied **RTP-1** in OLEDs as an emitter and fabricated OLEDs with a structure of indium tin oxide (ITO)/poly(3,4-ethylenedioxythiophene)-poly-(styrenesulfonic acid) (PEDOT:PSS)/polyvinylcarbazole (PVK):2-(4-biphenylyl)-5-(4-tert-butylphenyl)-1,3,4-oxadiazole (PBD):**RTP-1**/Ba/Al. The device showed EQE and current efficiency of 0.1% and 0.5 cd A^−1^ at 11 V, respectively. The performance was not satisfactory because the device architecture was not optimized and the PLQY measured in the same blend film (PVK:PBD:**RTP-1**) was only 2%, which is much lower than that measured in the powder. The result demonstrated that the low rigidity of blend film cannot effectively suppress the competitive deactivation process of the excited triplet state. Although the device performance was poor, this work is the first attempt at using pure organic RTP materials as an emitter in OLEDs. Pure organic RTP materials then became a new choice for OLEDs after fluorescence materials, phosphorescence materials and TADF materials. It should be noted that the luminescence mechanism of **RTP-1** in the film has not been well-studied, and the device exhibited different electroluminescence spectra under various voltages, which indicates that the device performance could be further improved by using proper host matrix and device structures.

Later in 2013, Chaudhuri et al. developed two pure organic RTP materials **RTP-2** and **RTP-3** (Figure 2) and their OLEDs were fabricated with a structure of ITO/PEDOT:PSS/N,N'-bis(3-methylphenyl)-N,N'-diphenylbenzidine (TPD)/PVK:**RTP-2** (or **RTP-3**)/PBD/CsF/Al (Chaudhuri et al., 2013). The electroluminescence spectra showed two distinct peaks, 560 and 690 nm for **RTP-2**, and 630 and 760 nm for **RTP-3**. Such dual emissions contain both singlet and triplet transitions of the molecule under electrical excitation and reveal a long-lived triplet afterglow. However, **RTP-2** and **RTP-3** showed low PLQY of 4.6 and 1.3% and their corresponding device also showed very low EQE of 2.54 × 10^−4^ and 5.58 × 10^−5^, respectively. In this work and subsequent work, the authors studied the photoluminescence properties of these RTP materials in doped polymer films in detail (Ratzke et al., 2016). Through characterization and attribution of different emission peaks in the spectra, the authors provided a new understanding on the behavior of RTP materials in film, which is useful for RTP materials to be applied as emitters in OLEDs.

In 2016, Kabe et al. dispersed **RTP-4** (Figure 2) into the host molecule of 3-(N-carbazolyl)-androst-2-ene (CzSte) and observed dual emissions of blue fluorescent and green phosphorescent under photoexcitation (Kabe et al., 2016). By using **RTP-4** doped CzSte as the EML, they fabricated an OLEDs with a device structure of ITO/4,4;-bis[N-(1-naphthyl)-N-phenyl-amino]biphenyl (α-NPD) (30 nm)/1,3-bis(N-carbazolyl)benzene (mCP) (10 nm)/ **RTP-4**:CzSte (1 %, 30 nm)/1,3,5-tris(N-phenylbenzimidazol-2-yl)benzene (TPBi) (60 nm)/LiF (0.8 nm)/Al (80 nm). The device emitted a blue emission with an EQE around 1% under the external electric current excitation. When the external electric current excitation was turned off, the device could still emit a green permanent emission with a lifetime of 0.39 s. The significant improvement of the device performance compared with the aforementioned two examples may arise from a better device fabrication method, i.e., thermal evaporation vs. solution spin coating, which provides more choices for host materials and other functional layers.

## Other Pure Organic RTP Materials Potential for OLEDs

The requirements for luminescent materials in OLEDs are often combined with the advantages of high PLQY and a short-excited state lifetime, in order to produce devices with a high efficiency and low efficiency roll-off. Screening by these requirements, there are a few pure organic RTP materials reported in the literature, based on which OLEDs are expected to have good performance.

In 2014, Koch et al. designed and synthesized a series of boron-based RTP materials (Koch et al., 2014). The photophysical results showed that the compounds **RTP-5**–**RTP-11** (Figure 2) have a high PLQY (29-104%) and a short-excited state lifetime (1233-5413 ns) that is suitable for OLEDs. In particular, the compound **RTP-7** showed a high PLQY up to 104% and a short-excited state lifetime of 1,382 ns in a diluted dichloromethane solution, as well as a high PLQY up to 118% in poly(methyl methacrylate) (PMMA). This indicates that the non-radiative transition in these compounds can be well-suppressed even in a less rigid atmosphere, leading to a high PLQY and short excited state lifetime comparable to phosphorescent complexes (Endo et al., 2008). However, OLEDs based on these RTP materials are yet to be fabricated and explored.

In 2016, Shimizu et al. reported **RTP-12**–**RTP-16** (Figure 2) as a new class of RTP materials (Shimizu et al., 2016). The crystals of these compounds showed photoluminescence under ambient conditions. Intermolecular interactions were observed in each crystal, which contributed to the restriction of intramolecular motion and suppressed non-radiative transitions. The excited state lifetimes were dozens of microseconds and the PLQY was 14 and 8% for **RTP-13** and **RTP-14**, respectively. Though these compounds were not emissive either in solution or in a doped polymer film, the short-excited state lifetime in microseconds is attractive for the high efficiency OLEDs, which deserves to be explored in more detail.

Later in 2016, Gutierrez et al. reported red phosphorescence from **RTP-17**–**RTP-20** (Figure 2) at room temperature (Gutierrez et al., 2016). The photophysical properties of these compounds in deoxygenated cyclohexane are presented. The excited state lifetime of 2.8–5.4 μs is similar to that of traditional phosphorescent complexes, making them suitable for OLEDs. However, the PLQYs of this class of compound in a solution are very low (<1%). The reason is probably due to the presence of long alkyl chains. In the low-rigid solution, the twisting of the alkyl chain may lead to an increase in the non-radiative transition. Reducing the length of the alkyl chain or incorporating the luminescent molecules into more rigid host materials could upgrade the PLQY of these compounds.

In 2018, Huang et al. proposed a series of donor-acceptor-donor (D–A–D) compounds **RTP-21**–**RTP-24** (Figure 2) (Huang et al., 2018). The unsubstituted compound **RTP-21** exists in both equatorial and axial forms in the ground state, but the equatorial conformer prevails in the excited state. The changing in conformers lead to enhancement of RTP emissions with a high PLQY up to 71% in zeonex solid films. The excited state lifetime of 63.3 μs also indicates that the molecule is suitable for application in OLEDs. The phosphorescence quantum yield of **RTP-21** is the highest among all current reported RTP molecules, and highly efficient OLEDs are expected when using **RTP-21** as the emitter.

## Conclusion and Outlook

In recent years, a variety of RTP materials have been designed and synthesized, the color of which can cover the entire visible region (Li et al., 2018). However, the application of pure organic RTP materials in OLEDs is still in its infancy. The reasons are as follows: (i) The PLQYs of pure organic RTP materials in the thin film state tend to be very low. Compared with the phosphorescent complexes or TADF materials commonly used in OLEDs, the PLQYs of pure organic RTP materials are still at low levels, and the effects of non-radiative transitions are significant. Most pure organic RTP materials tend to exhibit high PLQY in crystals or at low temperatures, while their PLQYs decrease significantly in a solution or a thin film at room temperature. The reason is that the rotation and vibration of the molecules are suppressed in a rigid environment or at a low temperature, where the rate of non-radiative transition is greatly reduced; (ii) The pure organic RTP materials have low radiative transition rates and long excited state lifetimes. Most of the reported pure organic RTP materials have lifetimes in the order of milliseconds or even seconds, and RTP materials with short lifetimes of several microseconds have hardly been reported. The long-excited state lifetime may result in serious triplet-triplet annihilation, leading to obvious efficiency roll-off in OLEDs.

Though the current performance of pure organic RTP based OLEDs is poor, it can theoretically achieve 100% IQE like noble metal complexes and TADF materials, which provide more possibilities for high performance OLEDs. The design and synthesis of pure organic RTP materials with a high PLQY and a short-excited state lifetime, especially in the thin film state, is the key goal in this field, since the EML in OLEDs is often a spin-coated or vapor-deposited thin film. To achieve this, it is conceivable to introduce n electrons containing atoms and heavy atoms (Saigusa and Azumi, 1979) into the luminescent molecule to accelerate *k*_ISC_ and *k*_p_, to improve the PLQY of the RTP materials. It is also possible to introduce a more rigid host material or a full-deuterated host to suppress non-radiative transitions. Moreover, OLEDs architecture optimization, using proper functional materials for balanced charge transport, favorable exciton confinement, and efficient energy transfer is also critical in improving the performance of RTP based OLEDs.

## Author Contributions

GZ wrote the manuscript. ZL, ZB, and CH helped to revise the manuscript.

### Conflict of Interest Statement

The authors declare that the research was conducted in the absence of any commercial or financial relationships that could be construed as a potential conflict of interest.
